# Cryptographic Algorithms with Data Shorter than the Encryption Key, Based on LZW and Huffman Coding

**DOI:** 10.3390/s23177408

**Published:** 2023-08-25

**Authors:** Tomasz Krokosz, Jarogniew Rykowski, Małgorzata Zajęcka, Robert Brzoza-Woch, Leszek Rutkowski

**Affiliations:** 1 Department of Information Technology, Poznań University of Economics and Business, 61-875 Poznan, Poland; tomasz.krokosz@ue.poznan.pl (T.K.); jarogniew.rykowski@ue.poznan.pl (J.R.); 2 Department of Computer Science, Faculty of Computer Science, Electronics and Telecommunications, AGH University of Krakow, 30-059 Krakow, Poland; mzajecka@agh.edu.pl (M.Z.);; 3 Systems Research Institute of the Polish Academy of Sciences, 01-447 Warsaw, Poland

**Keywords:** short encryption key, information security, Huffman coding, LZW, entropy, privacy, compression

## Abstract

Modern, commonly used cryptosystems based on encryption keys require that the length of the stream of encrypted data is approximately the length of the key or longer. In practice, this approach unnecessarily complicates strong encryption of very short messages commonly used for example in ultra-low-power and resource-constrained wireless network sensor nodes based on microcontrollers (MCUs). In such cases, the data payload can be as short as a few bits of data while the typical length of the key is several hundred bits or more. The article proposes an idea of employing a complex of two algorithms, initially applied for data compression, acting as a standard-length encryption key algorithm to increase the transmission security of very short data sequences, even as short as one or a few bytes. In this article, we present and evaluate an approach that uses LZW and Huffman coding to achieve data transmission obfuscation and a basic level of security.

## 1. Introduction

When data are transmitted over the network, various measures are required to protect its many aspects, including confidentiality [1], integrity [2], availability [3], non-ambiguity [4] and, where applicable, also validity [5], accountability [6] and reliability [7].

Data security is generally ensured through the use of cryptography. We currently have a rich collection of cryptographic algorithms available. Traditional encryption algorithms operate on the input data, i.e., the *plaintext*, and the *secret* on which generating the encryption key is based. However, the implementation of the algorithms heavily depends on the hardware on which they are run, namely operating memory, processor clock speed, and transmission speed, as well as the form of power supply and, in some cases, the capacity of the power supply battery [8]. These basic resources are usually not a barrier in the case of personal computers, servers, graphics cards or tablets, and smartphones. All of them have enough computing power to execute the encryption method and obtain an encrypted form of the message. However, in this article, we are considering the systems in the domain of resource-constrained MCU-based connected embedded devices and IoT nodes. In such systems, the hardware peripheral support, computing power, memory, communication bandwidth, and energy resources are significantly limited. Examples of such platforms include the following MCUs: RP2024, which is used in a very popular Raspberry Pi Pico platform, or the STM32 series from ST Microelectronics, which are commonly used in industrial electronics. In general, many of the low-end and mid-end MCUs have no hardware-supported encryption peripherals. In these cases, it may be difficult or inefficient to use popular and widely available algorithms such as AES or 3DES. For such platforms, the hardware resource demands to obtain the result—the ciphertext—would be unacceptable. Moreover, performing the algorithm itself would reduce the expected time of their battery operation time due to the greater demand for energy. Importantly, some devices send messages of length from single bits up to bytes. Thus, using an algorithm with a key length of, for example, 256 bits is against the principle of a key size similar to the size of an encrypted block. This is a case of simple devices with a primitive structure that send a small volume of data for further processing (e.g., one-way transmission, only sending data like temperature or pressure value). In order to effectively achieve the goal and ensure the required security aspects, they must be equipped with dedicated, non-standard expensive solutions that significantly increase the use of available resources—mainly battery energy. Designing the new cryptographic algorithms for this specific group of devices is a very demanding challenge. In this paper, we propose a new method for short data encryption based on the non-standard application of two compression algorithms, namely the Huffman algorithm and LZW. These compression algorithms are widely used for shortening the data length without losing data meaning (information contents). However, so far, these algorithms were not applied for data obfuscation. We propose to secure some parameters and data structures for these algorithms to act as an equivalence of an encryption key. As the length of the compressed data is not limited (neither the lower nor the upper value), we may encrypt very short data (in theory—even a single bit) with an encryption strength comparable to the classical cryptographic algorithms. We also propose some mechanisms, based on random noise to obfuscate the slowly evolving sensor values (such as a temperature value) so that the subsequent transmissions differ significantly. Due to the compression factor, the added noise is not increasing the overall data length.

It is important to point out that the described solution does not aim at replacing any existing algorithm which is in use for typical data and key lengths. Our solution is, however, devoted to the application area which typical encryption solutions cannot address or for which classic algorithms are inefficient or just inconvenient to use.

The organization of the article is as follows. We start with a brief summary of the current state of the art in Section 2. Then, in Section 3, we present the overview of the proposed solution. Section 4 is devoted to theoretical issues concerning compression algorithms and their application in the subject of data encryption. Section 5 presents the technology-agnostic example of an implementation procedure necessary to be undertaken using the algorithms used in the described solution. For clarity, the procedure is presented using a set of sample data. The practical evaluation of the proposed procedure is described in Section 6. Results of the implementation are obtained from an example implementation in real hardware. That section also discusses selected security aspects. Finally, Section 7 contains a discussion of the achieved results and the usefulness of the solution.

## 2. Related Work

Specifying and describing individual steps and subsequent iterations of a cryptographic algorithm should be widely known, as they should all be designed according to the Kerckhoffs principle [9]. This principle says that a cryptographic system should be secure even when all the details of its operation—except for the decryption key—are known. The encryption/decryption key is the decisive parameter, having an influence on how easy it is to obtain the plaintext from the ciphertext in whole or in part. If the encryption and decryption keys are the same, we speak about symmetric cryptography; otherwise, asymmetric solutions apply. An example of a symmetric algorithm is, e.g., 3DES [10] or AES [11], where the recommended key length is up to 256 bits. In turn, with regard to the typical asymmetric RSA algorithm [12], a key length of 1024–4096 bits is recommended. Authentication is ensured by, for example, the HMAC mechanism [13], where the typical key length should be 224–512 bits when using the SHA-2 algorithm [14]. As we see, in the case of small sensors, the encryption key length is usually substantially much longer than the encrypted data, forcing a transmission of unnecessary information or even ultimately preventing the practical applications for limited hardware.

Also, widely used block ciphers [15] may not be useful in the described situations. Padding a very short message to the full block size would contribute to the unnecessary transmission of redundant data. Moreover, such an approach is inefficient in systems with limited caching capacity and in cases where chunks of data need to be processed as they are received. In the scope of this article, only one bit, one byte, or another small chunk of data is encrypted at a given time. In such use cases, the stream ciphers [16] will also not prove efficient due to the length of the payload. Moreover, a pseudorandom number generator (PRNG) [17] will be required to ensure their correct operation. The PRNG provides necessary random values on an ongoing basis. It is always the element of stream ciphers that is their weakest and the first aspect to be broken due to, e.g., repetition cycles. In addition, specific situations in which each bit is encrypted separately require additional synchronization mechanisms.

## 3. Overview of the Proposed Solution

In order to solve the problem, the authors of the article propose a new approach enabling short data encryption with a longer key. In this case, the innovative concept is based on Huffman coding [18,19] in combination with LZW [20,21], which will ensure the desired level of security for a dedicated group of devices. The LZW algorithm, based on the detection of correlations between successive characters, generates a dictionary with codes representing symbols and their concatenations. Then, based on the dictionary data, the Huffman algorithm implementation procedure is started, during which a code is determined for each index coded in the first stage, with a length inversely proportional to the frequency of its occurrence. This means that symbols and their concatenations, represented in the form of indexes often found in the message, are ultimately represented by much shorter codes. The result is not only a significant reduction in redundancy, a reduction in the number of transmitted packets, and the space needed to save data, but also a binary tree used for data compression. Because we assume that an encoded representation of the binary tree is used as an equivalent to an encryption key, it is possible to encode messages of any length, including ones that are shorter than the key.

## 4. Materials and Methods

In the domain of digital representation, it may happen that the information to be stored or transmitted has redundant, often repetitive, chunks of data. It usually results in reserving more bits or blocks of data than are actually needed to represent the data unambiguously. This is the reason for the undesirable phenomenon of data redundancy, which can be eliminated or reduced by compressing the data. Compression [22] makes it possible to encode information in a way that will contribute to saving resources, including the required operating memory or occupied disk space, and will also allow the same information to be sent in a much smaller number of packets and in a shorter time.

In this article, we consider the lossless type of compression in which the compressed data can be restored to its exact, original form.

### 4.1. The LZW Algorithm

The LZ77 algorithm was dedicated to solutions where we assume that repeating symbols occur within a short codeword distance. The improved LZ78 algorithm alleviates that limitation. In this case, compression is as straightforward as substituting symbol strings with indexes in the dictionary that stores them. Lossless compression is achieved by substituting a reoccurring sequence with a reference to the position of its copy held in the dictionary. The reference is a tuple (i,l,c), where o≥0 is the *offset* position of the copy in the dictionary (in our case counted backward from the end of the dictionary), l≤0 is the *length* of the sequence and *c* is the next character after the reoccurring sequence in word *M*. The dictionary can be provided externally and can be expanded as needed. The data that have already been processed can be removed from the dictionary. The place of the triple from the LZ77 algorithm is replaced by the pair (i,c), where *i* is the best-fit index and *c* is a character or a symbol ending the match.

The result of further work on the LZ78 is the LZW algorithm proposed in 1984. The principle of its operation is very similar to those previously mentioned, with the difference that instead of the pair (i,c), only the *p* position of the longest match is encoded. Notably, the obligatory step is to include all the symbols of the alphabet in the dictionary. This requirement is due to the impossibility of encoding a symbol when encountered when it is not in the dictionary. In this way, the dictionary is ultimately represented by concatenating the encoded dictionary element and the *s* symbol following it. An additional, undoubted advantage is also the lack of a need to remember and transmit the dictionary. Instead, the dictionary can be reproduced by another node based on the encoded form of the message. The universality of the LZW algorithm should also be mentioned because its implementation is unrelated to any compressed data conditions. The compression method depends on the source, i.e., the message. It means that the algorithm is not only dynamic but also adaptive. The advantage of LZW over the two previously mentioned algorithms manifests itself in the form of a shorter output, as only the index of the found word is given. This does not mean, however, that the LZ77 and LZ78 will not be used in other dedicated solutions designed for a specific purpose. The result obtained with their help may be completely sufficient within a specified application area; therefore, when specifying functional requirements, it is also worth considering them.

### 4.2. Huffman Algorithm

One way to encode characters is to use fixed-length codes, where the length of each encoded symbol will always be the same. No matter how often an element occurs in relation to other elements, the lengths of all codes will be identical. In addition, equal weights guarantee very good results when the number of characters is small. A greater degree of compression is achieved with variable-length codes implemented using binary trees. To visualize this, let us consider, for example, the alphabet A={a,b,c}. There is no need to reserve a byte of memory to represent any symbol because 2 bits is enough. Therefore let us assume the following mapping: *a*—11, *b*—00, *c*—01. To encode the text abbccbba, consisting of 8 symbols, 64 bits should be reserved in the standard solution. Using fixed-length codes makes it possible to shorten the transmission to 16 bits, which is a compression result of seventy-five percent.

It can be seen that it is much better to assign short codes to frequently occurring symbols. This marked the beginning of an alternative to the described case. In the presented algorithm, the first step is to count the occurrences of each symbol in the input data. This means that each character has a counter associated with how many times the symbol occurs in the message. The most common characters are represented by short codes. On the other hand, longer codes are associated with less frequent symbols. Therefore, the length of the code is inversely proportional to the frequency of the symbol.

The input to the algorithm is a set of symbols of size n and weights $W=(w_{1},w_{2},\ldots ,w_{n})$ that are usually proportional to the probabilities $w_{i}=weight\left(a_{i}\right)$, i∈{1,2,…,n}. As a result of the implementation of the algorithm, a binary code with the minimum expected length of the code word is obtained, i.e., the equivalent of a tree with the minimum weighted length of the path from the root $C\left(W\right)=\left(c_{1},c_{2},\ldots ,c_{n}\right)$. This means that the code is a tuple of binary codewords, where c+i represents the code for $a_{i}$, for i∈{1,2,…,n} [23,24]. For the data generated this way, the next step is to create a binary tree built from the leaves to the root and label all edges with the values 0 (left) and 1 (right). This way, it becomes possible to unambiguously interpret a message fragment at any stage of data processing stage.

### 4.3. Combination of LZW and Huffman Algorithms

In the presented solution, we have decided to use a combination of Huffman and LZW algorithms. The first of these is oriented to detect and compress data with a focus on the frequency of occurrence of individual symbols. Unfortunately, when used alone, the algorithm does not allow the detection of correlations between successive characters. This is provided by the LZW algorithm. This way, the initial redundancy is reduced to a much lower level.

The proposed solution includes not only data compression and protection but also additional authentication. Linking the message with the node’s identifier and encoding it as a whole allows for the introduction of additional verification mechanisms. In this way, we obtain unambiguous confirmation of who is sending the data, proven by the ability to encode the data correctly. If the data sender knew the key, they were able to carry this out, so they are who they claim to be. For the purpose of authentication, a mapping structure can be stored in the database (directory) in which the association of the key with the address will be stored. As a result, detecting the sender’s address determines the decoding key from the set, which is part of the authentication process. When we replace the identifier with any right (e.g., a *read* or *write* permission), we obtain the possibility of authorization. A necessary step in the case of the described implementation is the need to agree on the format of the message or packet in advance.

The implementation of the two presented algorithms is popular, e.g., it takes place in a Linux/UNIX system when the *compress* method is called. This combination was also used in classic operating systems, in the PKZIP program, and in the WinZip application. Therefore, it seems justified to use these two algorithms in the domain of resource-constrained systems to ensure compression as well as protection.

### 4.4. Application of the Algorithms

**Figure 1:**
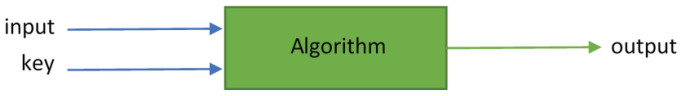
Typical application of an encryption block.

Encryption algorithms require additional data to ensure proper operation, e.g., confidentiality and data integrity. In addition to input, encryption algorithms typically require a key to be provided. For data security, the length of the key must be close to the size of the block of plaintext that is being encrypted. The classic approach to the case is shown in Figure 1. This way, if we need to encrypt a shorter data block, we need to pad that information with random data up to the minimum block length supported by the encryption algorithm.

The data transmission should be minimized in systems that use very low power transmission and resource-constrained hardware. Examples of such systems are low-power IoT devices or wireless sensor networks in which a single sensor is required to operate for up to a few years on a single battery. Such systems often transmit very little amounts of data sparsely in time. In such cases, padding very short messages to align their length with the key will be inefficient. Therefore, there is a gap in the subject of data security in the domain of resource-constrained sensors, and the presented solution intends to fulfill that gap.

**Figure 2:**
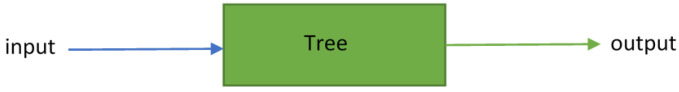
The proposed encryption scheme uses a binary tree as the method of representation of data compression.

The data protection proposal presented in the article uses a combination of two data compression algorithms with different characteristics. The first of the algorithms, LZW, is a kind of dictionary compression algorithm. It allows us to detect strings in the text that are often repeated in the input data. Each detected and already encoded part of the message in each subsequent iteration is extended by the next symbol that ends the longest match. In this way, after processing the input data, a dictionary is obtained containing a mapping of a string of several, even a dozen or so, characters for the index identifying it. On the basis of frequently repeated symbols (indices), the frequency of their presence in the message is determined. This allows frequent indices to be assigned shorter codes than less frequent indices, based on the Huffman coding method. As a result, after applying a combination of these algorithms, a binary tree is generated. The edges of that tree are then marked with labels. It enables unambiguous interpretation of the value (leaf) during encoding or decoding. In our case, it can be perceived as an encryption and decryption of the message. The size of the tree, i.e., the final number of leaves (encoded indexes), is determined on the basis of the contents of the LZW dictionary. If such a dictionary is kept secret, this means that the binary tree is also a secret, being an equivalence of an encryption key in this case. Therefore, we propose a new data protection scheme, presented in Figure 2.

## 5. Sample Application Procedure

This section presents the procedure to be taken in order to properly implement the algorithms. The first necessary step is to determine the relationships among the symbols, based on the processed set of symbols. The occurrence of repetitive strings in successive iterations expands the resources of the dictionary, in which encoded values are stored under successive indexes. They are created as a result of a concatenation of the longest, identical match of the currently processed fragment with the one already saved, with the symbol that ends this match. In this way, a dictionary is generated, by means of which an increasingly long string of characters is represented by an index (LZW algorithm). In the second step, the dictionary data are evaluated against the index frequency. On the basis of this size, their weights are determined, which ultimately determines the location of the leaf. The most frequently appearing indexes will obtain the shortest codes, while for those to which references are less frequent, the path leading from the root to the leaf will be longer (Huffman’s algorithm).

### 5.1. Analysis of an Example of Huffman Algorithm Application

**Figure 3:**
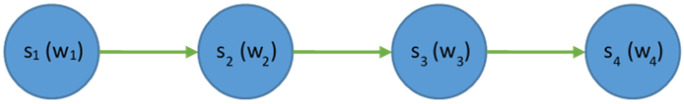
The list of nodes sorted ascending by their weights ($w_{1}\ldots w_{4}$).

The effect obtained by running the Huffman algorithm for the input data is the assignment of short codes to the most frequently occurring characters in the plaintext message. This means that characters appearing less often receive longer codes, so the length of the code is inversely proportional to the frequency of the symbol in the message. For the purposes of presenting the theoretical background necessary to explain the issue, let us assume that alphabet A is built of four symbols, i.e., $A=\{s_{1},s_{2},s_{3},s_{4}\}$. Each symbol is associated with its weight, i.e., the frequency expressing the probability of its occurrence, i.e., $w=(w_{1},w_{2},w_{3},w_{4})$. The first step of the Huffman algorithm is to create a list of tree nodes. The nodes will eventually take the form of leaves in the binary tree to store the encoded character and its frequency (probability of occurrence) in the text. This list is organized in ascending order by weight (Figure 3).

**Figure 4:**
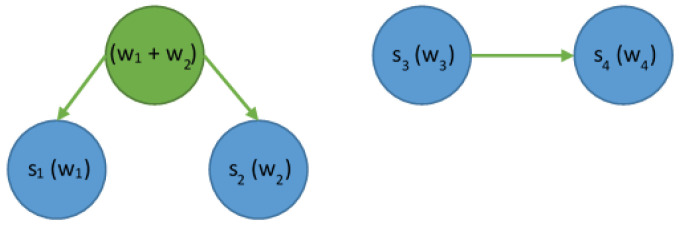
The list of nodes after merging the two lowest-weight nodes.

The first two nodes with the lowest weights are merged, and a new node will be created. We store the sum of weights, i.e., the probabilities of symbol occurrences in it. Such a node does not represent a specific symbol and it is not a leaf; therefore, it should be treated as an internal node. The node created in this way is then inserted into the list of nodes while considering the ascending order of weights (Figure 4).

**Figure 5:**
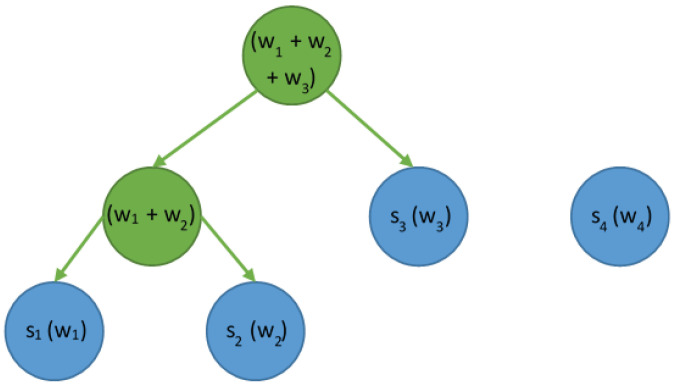
The list of nodes after merging the subsequent two nodes.

The operation is repeated. Two nodes with the lowest weights are selected, which will then be merged. In this case, we take the node obtained as a result of the merge in the previous step and also the one with the symbol $s_{3}$ and the weight $w_{3}$ (Figure 5).

**Figure 6:**
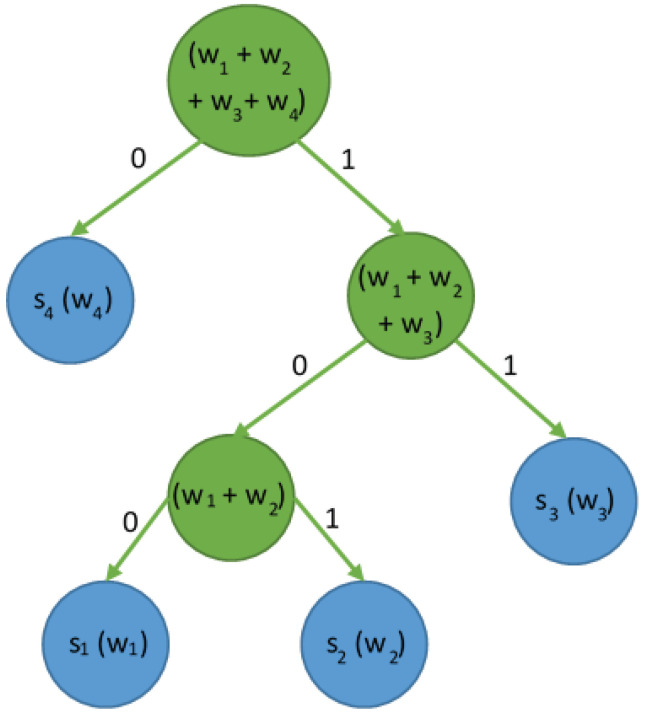
The resulting Huffman tree with edges labeled with binary values.

At this stage of the algorithm, there is one node that needs to be merged ($s_{4}$), so the operation should be performed one last time. Whether $s_{4}$ is on the left or right depends on the symbol weight value. According to the obligatory rule, nodes with a lower frequency value are on the left. When the finished tree is generated, all edges should be labeled. The rule is to mark the left edge with the value 0 and the right edge with 1. The result is shown in Figure 6.

The generated tree and given labels enable the reading of codes representing specific symbols; here, $s_{1}=100$, $s_{2}=101$, $s_{3}=11$, and $s_{4}=0$. For example, if a message *K* is represented as $K=s_{3}s_{4}s_{1}s_{2}s_{3}s_{4}s_{3}$, then the encoded message will be represented as 11010010111101011.

### 5.2. Analysis of an Example of LZW Algorithm Application

The LZW algorithm provides lossless compression and uses dynamic dictionaries. As mentioned earlier, as a result of the implementation of the method, subsequent inputs are encoded using the pair (i,C). The value of *i* is the index of the beginning of the best match, and *C* is the symbol that ends it. After each match, a string is added to the dictionary, which is a concatenation of the matched string and the character immediately after it. This means that subsequent dictionary entries are inserted as a result of combining the best match of the currently processed text and the character that immediately follows it. According to Terry Welch’s work [20], there is no need to encode the two elements of the pair achieved in the initial initialization of the dictionary. This is related to placing all the symbols that make up the alphabet in the dictionary at the beginning of the encoding. When the currently processed fragment is not present in the dictionary, the best match found is concatenated with the symbol that ends it. The text obtained in this way is inserted into the dictionary under a new, subsequent index, while the symbol ending the match becomes the first one from which the next match will take place.

Let us have an alphabet consisting of four symbols: $A=\{s_{1},s_{2},s_{3},s_{4}\}$. The dictionary is initially empty, so there is a need to save them under subsequent indexes: $s_{1}$ at index 0, $s_{2}$ at index 1, $s_{3}$ at index 2, and $s_{4}$ at index 3.

Let us assume that the message to be sent is $s_{2}s_{3}s_{2}s_{1}s_{2}s_{3}s_{2}s_{4}$. The first symbol in the message, $s_{2}$, is found at index 1. There is no coded fragment $s_{2}s_{3}$ in the dictionary, so a single symbol is the best match. A new index, index 4, is inserted into the dictionary, which will represent the phrase $s_{2}s_{3}$. The symbol that completes the match becomes the first one from which the search for the next best match begins. The symbol $s_{3}$ is in the dictionary, but $s_{3}s_{2}$ has not yet been encoded. This means creating a new record in the dictionary with index 5, which points to the text fragment $s_{3}s_{2}$. The presented procedure is repeated until all the symbols constituting the input data are processed. For a sample set of symbols, the target result is shown in Table 1.

After the LZW encoding stage is completed, it is possible to read the encoded form of the message. In this case, the message $s_{2}s_{3}s_{2}s_{1}s_{2}s_{3}s_{2}s_{4}$ will take the following form: 121041.

It is worth noting that the coding process started with completing the dictionary. Once the regularities or repetitions that occur based on the text have been recorded, then the degree of compression is much higher. For example, if a fragment identical to the presented input data $s_{2}s_{3}s_{2}s_{1}s_{2}s_{3}s_{2}s_{4}$ was already encoded in the dictionary, then only one index would be enough to represent it, e.g., 23.

After the encoding process is completed, sending the result does not require attaching a dictionary to the message. Based on the encoded content and the initial form of the dictionary, the recipient can rebuild it and obtain a definitive version of the message.

### 5.3. Huffman and LZW Algorithms Combined

In the previous chapters, the typical application of LZW and Huffman algorithms was assumed, i.e., the individual building of data structures for the purpose of encoding one specific input data. In contrast to the traditional approach, in the proposed solution, we use the LZW and Huffman algorithms for theoretically arbitrary input data. For this reason, the data structures of these algorithms should include not only selected but all possible words from a given alphabet. On the other hand, it should be taken into account that some words (symbol combinations in the input) appear more often, so it is worth optimizing these structures for them. Therefore, a compromise has been adopted in the following solution. In the first step, structures are created for arbitrary combinations of alphabet symbols with a maximum length of *N* symbols. The number *N* is selected in terms of the capabilities of the microcontrollers in which the algorithm will be run. For N=2 and an alphabet consisting of characters that allow writing any number (14 symbols described below), the final table of the implementation of the combination of algorithms has about 700 elements. The length of this table grows exponentially with the increase in the number *N* and the length of the alphabet; therefore, assuming N=2 seems to be the optimal solution in practice.

Random occurrence counters in the input string are recorded for all generated words (clusters of symbols). This approach allows generating a random assignment of the probability of a given word, and thus, random data structures that become the equivalent of a randomly selected key of the encryption algorithm.

If we have a sufficiently long learning string, we can use it to optimize data structures, specifically the dictionary of the LZW algorithm. The words detected in the training string are processed according to the basic LZW algorithm, increasing the probability of occurrence generated in the previous step. The weights of the instances in the training sequence are selected so that the probability values considered in this way are slightly higher than the randomly generated ones. This allows some optimization of the length of encrypted messages while maintaining the randomness of the “encryption key”. The condition of using the training string for this purpose is its concealment. However, this string is not interceptable without breaking the algorithm or accessing additional internal data, so hiding it is a relatively simple task.

We assume that in the sample transmission, we want to hide the transmitted numerical data. Let us assume that alphabet *A* consists of symbols that allow one to write a number in any commonly accepted format, A={0,1,2,3,4,5,6,7,8,9,+,−,.}. In order to hide values that are repeated in successive transmissions (e.g., for slowly changing temperature data), we add some noise characters $S=\{@,\#,\$,\%{,}^{,}\&,*,\left(,\right),\_,=,!,\}$. The noise is added to the representation of each number sent, with 50% extra characters of a random value and in random positions, and removed when the message is decrypted at the destination. For example, the number 1.23 is padded with noise, e.g., @1!.23, 1$.(2!3, or similar.

For the maximum length of the input words represented in the dictionary with the assumed N=2, we generate and save to the dictionary all possible character combinations, supplementing them with a randomly selected number of repetitions. Such combinations take into account both proper alphabet characters and noise.

If we have the training sequence with noise, we use it in the standard form of the LZW algorithm. We create a tree for all possible combinations of substrings, so we constrain the length of the substring to make the tree a reasonable size. We randomly select the number of repetitions (i.e., the probability of occurrence) for each subsequence. After creating all the combinations, if we have a training string, then for each combination detected in the training string, we increase the number of repetitions for that combination by 10%. In this way, we increase the “priority” for those combinations that are more likely—they will potentially be encoded with fewer bits, with the accuracy of random selection when generating the whole at the very beginning. The generated substrings can have a maximum length of M=2·N due to the limited amount of memory in typical microcontrollers. In the example implementation, increasing the number of repetitions by 10% was assumed to gain the frequent repetitions of some substrings in order to not falsify the random order of them as computed in the first stage. For all detected substrings of length greater than *N* that have not yet appeared in the dictionary, we assume that they are not directly represented in the data structures for the LZW algorithm. This causes a division of such longer substrings to the substrings already represented so as not to overload the restricted memory of a microcontroller.

The second step is to create a binary tree based on the output data of the LZW algorithm and to implement the Huffman algorithm. When virtually any string of alphabetic characters can appear in the input, optimization of the Huffman tree for specific symbol occurrences is not needed. Instead, we can generate this tree based solely on randomized values for the number of repetitions for each element of the LZW array. We should carry this out in such a way that the tree is reasonably “fair” for any combination of input symbols, including noise. For this reason, we modified the classic Huffman algorithm, resigning from changing the order of nodes of the already generated sub-tree. The algorithm used is as follows.

1. Move all elements of the LZW array to a temporary repository marking them as “final” in the Huffman tree. The repository should be organized as an array.
2. Repeat steps 3–4 below until the temporary repository contains only one item.
3. Find the pair of two items in the temporary repository that have the lowest repeat count values. If several items have the same count value, select the item that is closer to the top of the table.
4. For each pair found, add the elements of that pair to the final array. In the temporary repository, generate a new item that is the “parent” of this pair. This parent element, which is described by the sum of occurrences of both elements, also contains the identifiers of these elements as “left” and “right” children. Remove both members of the pair from the temporary repository.
5. The last item from the temporary repository is the root of the Huffman tree. Add it to the end array and mark it as the start element of that array.

The final array generated by the algorithm is statistically balanced, i.e., all leaves of the derivation tree differ statistically by at most one depth in the tree. This is due to the fact that these elements are taken into account at the beginning of the algorithm because their occurrence counts are relatively small compared to the elements that were created as pairs in later steps. For the aforementioned 702 elements of the LZW table, the number of these levels is 11, and the number of vertices is about 1400. This means that each digraph is encoded using 10 or 11 bits, i.e., about 5 bits are devoted to encoding one character. This means a compression of about 70%. This result is definitely worse than when matching each input string separately. However, such a result is satisfactory from the point of view of the assumed goals, i.e., to obfuscate any input string. Statistically, we obtain even better values for substrings from the training string with a maximum length of 4. For these substrings, the effective number of encoding bits is 2–3.

Then, labels have been applied to all edges in the generated tree. The tree is represented as the final array. The labels will allow unambiguous interpretation when reading as well as when encoding the message. By navigating from the root to the leaves representing a particular symbol, we can obtain the encoded form of the message. This works in the same way as the classic Huffman algorithm. Such an array can be sent in the OTA mode to the non-volatile memory (e.g., flash) of microcontrollers, once or periodically at selected times. In this way, we can simplify to a minimum the code needed to use the described approach in end devices, i.e., network nodes. The generation of the array itself can be performed by a unit with more computing power, e.g., a computer. On the other hand, microcontrollers in network nodes use a ready-made representation of the array.

As one may see, the basic changes introduced to the original algorithms are as follows. For the LZW algorithm, the set of elements is related to any combination of two symbols from the input, as well as single symbols (all characters in the alphabet). The number of occurrences of each of the elements is initially a random value. Thus, such a set is not optimized to encode any given message. On the contrary, the set can represent many different messages; however, these messages are not in the maximum compression method. For the Huffman algorithm, instead of processing one message, we consider all the above-depicted LZW elements to deal with any message. Instead of generating an optimum Huffman tree for a single message, as with the original solution, we can process any message (however, in a non-optimal way). As a result, the final compression ratio is not optimal; however, the system is ready to process any string of input characters with a reasonable compression ratio.

The result of applying the Huffman coding algorithm to the data obtained as a result of the completion of the LZW algorithm is the processed data from the input string. The encryption key, in this case, is a binary tree, which enables effective processing of input data of virtually any length without statistically increasing the number of transmitted data despite the addition of approx. 50% noise, and sometimes even obtaining a slightly shorter output string than the input.

The algorithm for using the newly generated tree to parse the input string is the following.

1. Set the current input string buffer position to zero.
2. If at least two characters are remaining, starting at the current position to the end of the input string, form a temporary substring composed of a pair of the next two characters and advance the position by two; otherwise, form the substring of the last character in the input string.
3. Find the Huffman + LZW tree node connected to the substring and generate output based on this node by adding the node-represented set of bits.
4. Continue steps 2–3 until the whole input string is processed, and the current position in the input buffer points to the string’s end.
5. If the number of output bits is not a multiple of eight, add a certain number of 0 bits to complement the output.
6. Group each 8-bit part to represent it as a byte.
7. If the output is to be sent by a text channel, convert the bytes to some printable characters using some well-known algorithms such as Base64; otherwise, send the output as a binary string.

The algorithm for parsing the received string to restore the original message is the following.

1. Convert received string to a bit array, using Base64 text-to-binary decoding if necessary.
2. Set the current input position to zero.
3. Set the current tree node to the root.
4. Read the bit value from the current input position. Find a direct child of the current node based on this bit value. If the selected child is a leaf of the tree, add the set of two characters (a single character for some nodes) to the output and return to step 3; otherwise, advance the input position by one and repeat step 4.
5. Execute steps 3–4 until the current input position points to the end of the input string, or less than 8 bits remain at the input and are all zeros.

## 6. Evaluation and Results

The described encryption and compression algorithms have been successfully implemented in practice on two very popular hardware platforms: (1) the STM32L476 with ARM Cortex-M4 microprocessor on STM32 Nucleo-64 board manufactured by ST Microelectronics and obtained from a local distributor in Poland. with its core operating at the frequency of 48 MHz and (2) using the ESP32 WROOM-D hardware platform running at 240 MHz main clock frequency. The test program was written in C++ and it was compiled for each platform using the GNU C++ Compiler (g++, version MinGW-W64 v. 12.2.0) from Arduino IDE and framework. The time and performance measurements have also been implemented in the test program by using the available built-in timers. The test program performed the following tasks while measuring the time of each of them:

- Generating an ASCII-encoded data stream or packet which is 1 to 16 bytes (characters) long.
- Applying noise to the stream according to the description in Section 5.3.
- Encoding–encrypting the stream with the proposed combination of Huffman and LZW algorithms using the previously generated binary tree as a key.
- Decrypting the stream.
- Removing the noise from the stream.
- Formatting a line of output data in CSV format, including the information of measured times and effects of noise application as well as encryption and decryption, then printing the information using a hardware serial port.

The test program performed 25,000 tests, as described above, and the packet length was selected randomly for each sample.

An example of the first lines from the CSV output data is shown in Listing 1.

**Listing 1.** A sample output from the terminal while running the test program—a header and the first 3 records of raw data are shown; new lines were added for readability.
No; String; Length[B]; String_noised; Length[B];

String_encoded_bin;

String_encoded_hex; Length[B];

Add_noise_time[us]; Encryption_time[us];

Decryption_time[us]; Remove_noise_time[us];

Total_time[us]

0; 390; 3; =390; 4;

101111100110010001110000;

EB4607; 3;

3; 135; 156; 2; 296

1; 2.3583; 6; (!2.3583; 8;

011111000101111110101001101111011110010000000000;

C7F59ADB4E00; 6;

4; 366; 389; 5; 764

2; 2803924.91; 10; #_2803*924.91; 13;

111000001011110111110111110101000100001001000110000110001001010000000000;

0EDB7F4D2464814900; 9;

7; 599; 629; 7; 1242


### 6.1. Compression Ratio Analysis

The presented solution provides compression as an additional feature; thus, we have analyzed the compression efficiency of the proposed solution.

**Figure 7:**
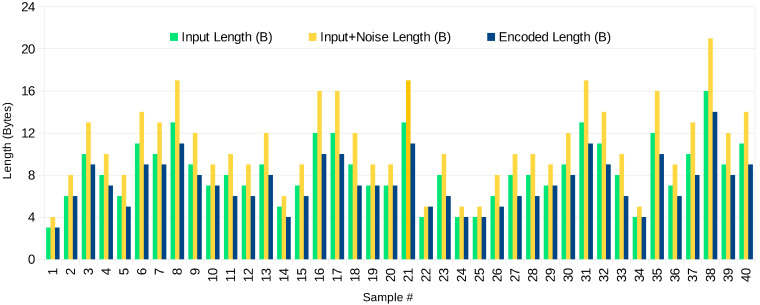
Comparison of length of packets at three stages of the processing algorithm: input (plaintext), input with noise, and in the encrypted-compressed form.

Data lengths for the first 40 test sample packets are shown in Figure 7. It can be noticed that the input data length is in most cases larger than the output data length.

**Figure 8:**
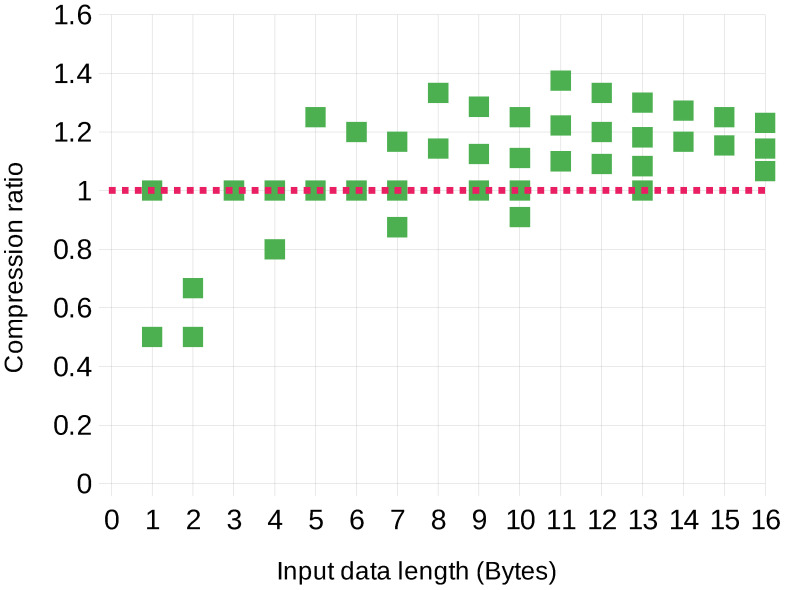
Observed compression ratios for various packet lengths. The compression ratio of 1 is marked for clarity.

Let us assume the classic definition of the *data compression ratio*: $DCR=\frac{USize}{CSize}$, where USize is the uncompressed data size, and CSize is the compressed data size. As can be noticed in Figure 7, in some cases, the “compressed” data has an inefficient DCR; however, many samples of longer packets have a better compression ratio. This is largely dependent on the packet length. Further analysis of this aspect is shown in Figure 8.

**Figure 9:**
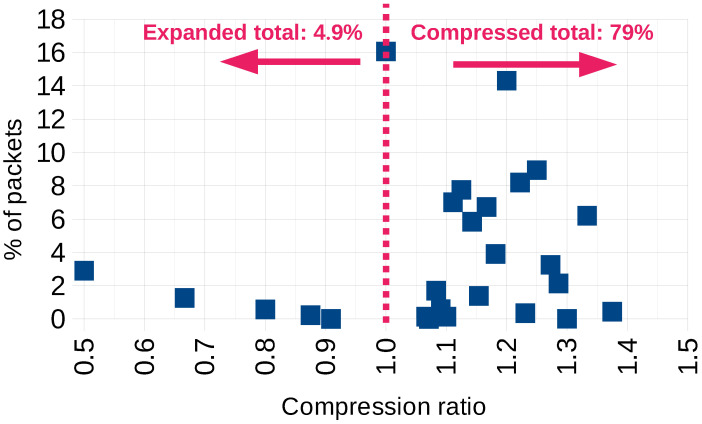
Distribution of compression efficiency (DCR). The compression ratio of 1 is marked for clarity.

The analysis of the percentage of packets that are efficiently compressed is shown in Figure 9.

### 6.2. Encryption and Decryption Time

**Figure 10:**
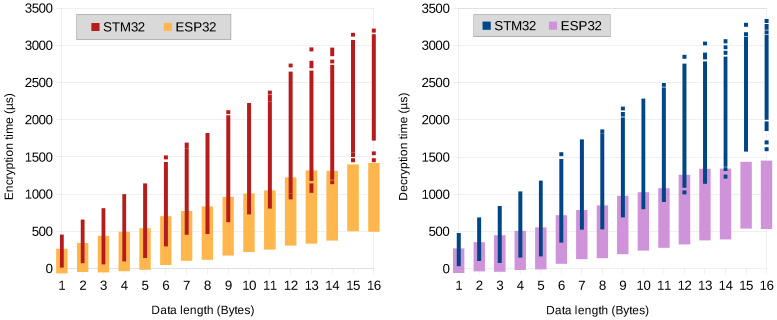
Encryption and decryption times.

The sample implementation performance has been evaluated on two different MCU platforms separately: on the ESP32 and STM32L series of microcontrollers. The resulting encryption and decryption times are shown in Figure 10. It can be observed that the resulting time depends predominantly on the encrypted data length and it is roughly linear both for the encryption and decryption process. For the tests, the core microprocessors of the ESP32 platform are clocked at the frequency of 240 MHz and the STM32L’s core frequency was set to 48 MHz. Both of these frequencies are commonly used values for these MCUs and are set by default in the Arduino IDE.

**Figure 11:**
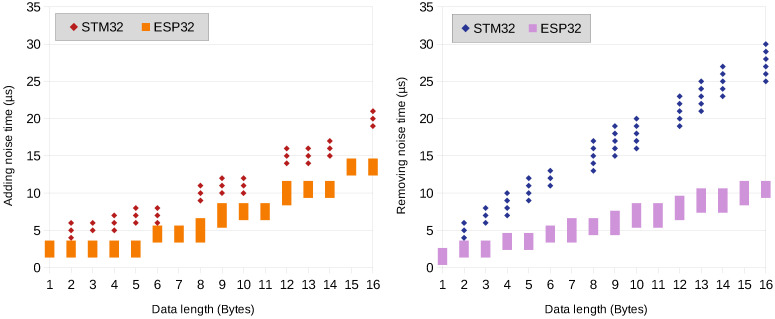
Adding and removing noise times.

The process of adding and removing noise is relatively fast and it also depends roughly linearly on the packet length. For the process of adding noise, the timing is linearly dependent on the packet length for lengths greater than 8 bytes. The adding and removing noise results are shown in Figure 11. The processing time has been measured with 2 µs resolution; therefore, the resulting times are also quantized to 1 µs value. The quantization, time resolution, and internal timer operation overhead may also be the reason why “adding noise time” may seem the same for the shortest packets of 1 to 5 bytes.

### 6.3. MCU Resource Usage

In the sample implementation, we are able to encode the ASCII characters representing basic numeric symbols. The array that represents the encoding tree is 1403 records long and each record is 20 bytes long. It results in the flash memory usage of 28,060 B for constant components required for encoding and decoding.

The RAM usage depends on the maximum length of the data to be encoded or decoded. We can assume that we need to store three buffers: for input data, noised data, and output data. The buffers need approximately three times the maximum length of a single packet or chunk. So, if we assume the maximum length of 16 bytes for a single packet, then the resulting static allocation will require approximately 50 bytes. Stack variable usage can be estimated at the level of two sizes of the buffer and some additional space for local variables and function calls. The overall memory RAM usage for processing 16-byte-long chunks of data would use about 80 bytes.

## 7. Discussion

In this section, we discuss the achieved results and analyze potential security considerations, scalability issues, performance, unique features of the proposed solution as well as differences with currently available encryption algorithms.

### 7.1. Security Considerations

Here, we discuss the possible ways of reading the correct value of the obfuscated message without the knowledge of the Huffman + LZW tree. The key parameter is the number of possibilities that exist for a certain length of the alphabet to generate the Huffman + LZW tree. It can be computed as follows. Let us assume that there are P=26 characters in the alphabet similarly as in the example described in Section 5. This includes basic characters and noise. Thus, the number of all possible pairs of the alphabet characters is $P_{1}=P^{2}=676$. As it is possible to encode also a single character, we have to add $P_{2}=P$ more possibilities, resulting in $L_{n}=\left(P_{1}+P_{2}\right)!=702!$ combinations. These combinations may be randomly placed at the Huffman or LZW tree in (Ln)! ways which, in practice, results in a number of the order of $10^{1500}$; thus, the probability of guessing the right tree is extremely low.

#### 7.1.1. Brute-Force Attack Considerations

A basic approach to a brute-force attack can be the following.

1. Guess the alphabet which is a subset of letters of *N* elements.
2. Guess the noise which is a subset of letters of *M* elements.
3. Generate a set of all possible ordered lists of subsets of one- and two-character substrings.
4. For each list from the set:
  (a) Generate Huffman + LZW tree using the algorithm from Section 5.3
  (b) Try to decode the message using the algorithm from Section 5.3
  (c) If failed, take the next element from the list

As one may see, a brute-force attack is not only based on some straightforward operations such as comparisons. On the contrary, for each loop of this attack, the complete Huffman + LZW tree must be generated, even to decode an incoming message of just a few bits.

If we can determine some more probable values of the input data, we may use a brute-force attack targeted only on selected pairs of characters, and thus selected parts of the tree. For example, if we know that the sensor transmits a room temperature *T*, the “most probable” values are in the range of 20.0 to 30.0 °C, so ΔT=30−20=10 °C. Thus, instead of guessing Ln different nodes, we obtain ΔT·Pa combinations for the pairs of types which include one digit and one noise character. We obtain the same for the pairs with one noise character and one digit, and 2·ΔT combinations for the pairs of two digits. This gives 540! combinations in total. Again, 540! stands for an extremely huge number of combinations, so even a partial brute-force attack is hard to perform in a reasonable time.

#### 7.1.2. Features which Make the Brute-Force Attacks More Difficult

One should also note another interesting observation. For typically utilized encryption algorithms, when we execute them with a wrong key, we obtain an output that is obviously wrong; for example, such a wrong output can be a random set of characters. Thus, it is relatively easy to determine if the brute-force attack iteration had been successful. However, while using the Huffman + LZW tree to decode the input bits, the attacker obtains something that may be similar to valid output data, but in such a case, it is still difficult to evaluate the real validity of the data. For example, for the same input, we obtain “22.1” as a result with one tree, and “17.5” as a result using another tree. Thus, additional semantic knowledge of the message content is necessary, such as which of the two temperatures above is more probable. As a consequence, the attacker cannot be sure if the guessed values are correct, even if the interpretation of the input generates no errors.

#### 7.1.3. Security Improvements

The explanation regarding the improvement of the obfuscation level may be necessary for the solution based on adding noise characters to the transmitted string or a number in the case of the example representing, e.g., air temperature or atmospheric pressure as read by a sensor. Let us analyze only the number of characters added as the noise—two noise characters for a four-digit-long numeric value. In such a case, we increase the number of possible combinations of each obfuscated message by (13·5!)·2=3120. This computation includes $P_{n}$ characters of noise, and a maximum of five places for each noise character and two noise characters per transmission can be inserted. However, after inserting the noise, we also provide bit-wise encryption of the noised message. As the number of bits for each encoded subset of characters depends on the place inside the tree this subset is registered, the attacker does not know which output bits are the equivalence of which input characters. Thus, the brute-force method must consider the number of the inserted noise characters and their neighboring characters. This fact raised the number of combinations by P, which is the length of the alphabet complemented with noise. Let us assume that a sensor, from which data are encrypted, is activated every second and it is sending the same value for a longer period of time. In such a case, we obtain the possible repetition of the same encrypted message in more or less a day, but still without increasing the length of the transmitted message. In such a case, the approximate length of messages will be about 5 bytes. Assuming one packet transmission in each minute, the possible repetition period can occur within a few months.

In our example, we use a restricted alphabet of $P_{a}=13$ characters complemented by $P_{n}=13$ noise characters, P=26. Thus, we generated the Huffman + LZW tree composed of $L_{n}=702$ leaf nodes (1402 nodes in total), resulting in 702! possible representations of the tree. If we add one single character and another one for noise, we obtain ${\left(P+2\right)}^{2}+\left(P+2\right)=812$ leaf nodes of the tree, resulting in 812! tree representations. As it may be seen, a very small enlargement of the alphabet results in a huge increase in possible configurations. However, these two numbers (702! and 812!) are so big that there is only a theoretical difference between them; regardless, a brute-force attack would take a very long time, even if generating a new tree every second.

### 7.2. Solution Scalability Discussion

We may also consider the extension of the tree which would represent triples (three-character-long substrings) rather than pairs of characters. Then, the number of leaf nodes of the tree is $P^{3}+P^{3}$ = 18,278. The maximum tree level is also increased from 11 to 16. The average compression factor slightly drops down to approximately 65%. However, such an extended tree would require about 200 KiB of non-volatile memory. This amount of memory is commonly available in many middle- and high-end microcontrollers, including ESP32, used by the authors for basic implementation. The ESP32 is equipped with usually between 2 and 8 MB of flash memory. However, for more resource-constrained microcontrollers, such as the STM32L476, with only 1 MiB of flash memory, such usage of flash memory could pose some problems. Moreover, the implementation could be impossible for smaller units, such as the STM32L0 series or for the popular ATmega AVR MCU series from Microchip. If we go even further and try to represent all four-character-long substrings with a Huffman + LZW tree, it would be impossible even for the mentioned resource-rich microcontrollers, such as the larger STM32 units and the ESP32 MCUs. To summarize, the chosen pair-based representation seems to be optimal for most application areas.

The presented concepts were successfully implemented and tested in the ESP32 series’ mid-end embedded microcontroller. The obtained results are promising and show that the developers can also incorporate these algorithms into devices equipped with low-end microcontrollers.

As shown in Section 6.3, the algorithm can be especially useful in resource-constrained and cost-effective wireless network nodes in which a microcontroller does not offer any means of hardware cryptography peripherals.

The concept of adding noise to the transmission can be interesting because it provides a basic level of security for very short and repetitive messages. It is especially useful for environmental condition sensors, such as digital thermometers. Even the slight amount of randomization and obfuscation makes the transmitted data less vulnerable to man-in-the-middle attacks.

As presented in Section 6.1, the data compression ratio depends to some degree on the length of the packet to be encrypted. We can assume that by *efficient compression*, we mean the DCR>1, while DCR=1 means *no compression*, i.e., the same input and output packet length, and DCR<1 is *inefficient compression*, which means that the “compressed” data could be larger than the plain text. The latter situation happens rarely and only in cases of the shortest packets, which may add just a few bytes to the original message length. In general, the proposed algorithm can be considered efficient because, for the streams of randomly chosen lengths from 1 to 16 bytes, it provides DCR>1 for 81.9% of the transmitted messages.

Discussing the proposed solution with classical encryption algorithms is challenging due to the difference in the application areas. First of all, the existing symmetric encryption algorithms require data length at least equal to the key length. Using typical encryption keys with the size of 2, 4, or even 8 kb, the minimum length of an encoded message has the size of approximately several hundred bytes. In our case, however, we are considering messages which are only a few bytes long. We cannot simply generate random bits to extend the short messages to long-enough ones because that would defeat the purpose of the presented method. The solution presented in this article concerns specific application areas. These are the reasons why the comparison with the commercially available solutions might not be meaningful in practice. The aspect of the application of noise to the slowly changing and repetitive data is orthogonal to the problem of the utilized encryption and decryption algorithms. This is also an interesting direction for possible future work on better adjustment of the noise-to-real data ratio in order to achieve more profitable use of the compression factor of our proposal.

## 8. Conclusions

In this paper, we proposed a novel approach for data obfuscation for very short data packets, even as short as a few bytes. Transfers of such short packets are the foundation for many sensor and actuator networks of the Internet of Things. These devices are very limited in battery time, computational power, memory, and radio bandwidth. To date, no efficient encryption techniques have been proposed for such application areas. Also, adjusting the classical encryption algorithms based on symmetric keys can be difficult, inconvenient and inefficient, mainly due to the length of the encryption key and the requirement for data length not to be shorter than this key. High memory and CPU power requirements are also worth mentioning.

In contrast, the proposed approach is characterized by minimum CPU and RAM requirements and reasonable ROM (flash) usage. The data payload can be as short as a few bits of data, while the typical length of an encryption key is several hundred bits or more. The article proposes the idea of employing a combination of two algorithms, initially applied for data compression. The algorithms act as a standard length encryption key algorithm to increase the transmission security of very short data sequences. Namely, our approach uses LZW and Huffman coding to achieve data transmission obfuscation. The Huffman + LZW tree, devoted initially to compression, is used as a secret key. The size of the tree is a counterpart of the encryption key length. As the tree may be used to process, in theory, any data packet with no restrictions on its size, we are able to encrypt and decrypt, with a significant level of security, even concise messages, in a very efficient way, using minimum resources of a microcontroller.

We implemented and evaluated the approach using two popular microcontrollers: Espressif ESP32 and STM L476. The test results confirmed the high efficiency and applicability of the solution.

## Figures and Tables

**Table 1:** An example of the LZW dictionary for the sample input values.

Index	Input	Output
0	$s_{1}$	-
1	$s_{2}$	-
2	$s_{3}$	-
3	$s_{4}$	-
4	$s_{2}s_{3}$	1
5	$s_{3}s_{2}$	2
6	$s_{2}s_{1}$	1
7	$s_{1}s_{2}$	0
8	$s_{2}s_{3}s_{2}$	4
9	$s_{2}s_{4}$	1

## Abbreviations

The following abbreviations are used in this manuscript:

ASCII	American Standard Code for Information Interchange
CSV	Comma-separated values
DCR	Data compression ratio
LZ77	Lempel-Ziv 1977
LZW	Lempel–Ziv–Welch
MCU	Microcontroller unit
RAM	Random Access Memory
